# Machine Learning Approach for the Outcome Prediction of Temporal Lobe Epilepsy Surgery

**DOI:** 10.1371/journal.pone.0062819

**Published:** 2013-04-30

**Authors:** Rubén Armañanzas, Lidia Alonso-Nanclares, Jesús DeFelipe-Oroquieta, Asta Kastanauskaite, Rafael G. de Sola, Javier DeFelipe, Concha Bielza, Pedro Larrañaga

**Affiliations:** 1 Departamento de Inteligencia Artificial, Universidad Politécnica de Madrid, Boadilla del Monte, Madrid, Spain; 2 Laboratorio Cajal de Circuitos Corticales, Universidad Politécnica de Madrid, Boadilla del Monte, Madrid, Spain; 3 Instituto Cajal, Centro Superior de Investigaciones Científicas, Madrid, Madrid, Spain; 4 Centro de Investigación Biomédica en Red sobre Enfermedades Neurodegenerativas, CIberNed, Madrid, Madrid, Spain; 5 Departamento de Psicología y Eduación, Universidad Camilo José Cela, Villanueva de la Cañada, Madrid, Spain; 6 Department of Neurosurgery, Hospital Universitario de la Princesa, Madrid, Madrid, Spain; Emory University, Georgia Institute of Technology, United States of America

## Abstract

Epilepsy surgery is effective in reducing both the number and frequency of seizures, particularly in temporal lobe epilepsy (TLE). Nevertheless, a significant proportion of these patients continue suffering seizures after surgery. Here we used a machine learning approach to predict the outcome of epilepsy surgery based on supervised classification data mining taking into account not only the common clinical variables, but also pathological and neuropsychological evaluations. We have generated models capable of predicting whether a patient with TLE secondary to hippocampal sclerosis will fully recover from epilepsy or not. The machine learning analysis revealed that outcome could be predicted with an estimated accuracy of almost 90% using some clinical and neuropsychological features. Importantly, not all the features were needed to perform the prediction; some of them proved to be irrelevant to the prognosis. Personality style was found to be one of the key features to predict the outcome. Although we examined relatively few cases, findings were verified across all data, showing that the machine learning approach described in the present study may be a powerful method. Since neuropsychological assessment of epileptic patients is a standard protocol in the pre-surgical evaluation, we propose to include these specific psychological tests and machine learning tools to improve the selection of candidates for epilepsy surgery.

## Introduction

Epilepsy surgery is effective in reducing both the number and the frequency of seizures, particularly in patients with temporal lobe epilepsy (TLE), a common form of intractable epilepsy [1]–[3]. Although epilepsy is associated with a variety of pathologies [4]–[5], hippocampal sclerosis is the most frequent pathological sign encountered in the resected temporal mesial structures of TLE patients [5]. Nevertheless, 30% of these patients continue to suffer seizures after surgery [6]–[11]. Thus, there is much interest in identifying the underlying causes of these surgical failures.

Several studies have investigated whether the outcome of epilepsy surgery can be predicted using clinical data, imaging techniques (MRI, PET, SPECT), electroencephalography functional tests (EEG, video-EEG), neuropsychology tests [11] or combinations of these approaches. Interestingly, TLE patients exhibit a variety of neuropsychological profiles that may change after surgery [12]. Such neuropsychological assessment can potentially identify the epileptogenic hemisphere [13], but this predictive potential has not previously been evaluated in detail in the case of the Rorschach test [14].

Since epilepsy is a highly complex disease that involves multiple factors, the predictive value of single variables has limited accuracy [15]. However, machine learning approaches can generate models that combine specific patient information to predict the outcome of the surgery treatment and, hence, help support clinical decision-making. Therefore, researchers have been trying to develop machine learning approaches as predictive tools for epilepsy. In particular, the application of artificial neuronal networks has been reported to reach a high accuracy (80% to 95%) in predicting the prognosis of epileptic patients [16]–[17]. Nevertheless, these artificial neuronal networks do not provide direct results on the relevance of individual variables or the inspection of the induced models.

This study proposes the use of a machine learning approach based on supervised classification and feature subset selection data mining to predict the outcome of epilepsy surgery. From a cohort of 260 patients from the epilepsy unit of the “Hospital de la Princesa” (Madrid, Spain), we selected those with, first, a well-defined hippocampal sclerosis after surgery and, secondly, a complete neuropsychological evaluation that included an assessment of cognitive-perceptive and emotional processes. Supervised classification data mining approaches were then used to generate computational models to predict whether a patient with TLE secondary to hippocampal sclerosis would fully recover following surgical intervention.

Data analysis revealed that the surgical outcome could be predicted with a high degree of accuracy using specific clinical and neuropsychological variables. In addition, certain variables were found to be uninformative in this prediction. One key finding for the outcome prediction was the importance of personality style, a parameter that refers to aspects of an individual's personality and their emotional functioning. We propose here that clinical evaluations for epilepsy surgery should include, in addition to the classical analyses, these specific psychological tests and the use of machine learning models as standard tools.

## Materials and Methods

### Patients

Patients were pre-surgically evaluated according to the protocol used at the “Hospital de la Princesa” (Madrid, Spain), as described elsewhere [8]. In all cases, written informed consent was obtained from all participants in accordance with the Helsinki Declaration [18]. The study and all protocols received institutional ethics approval by the ethical committee at the “Hospital de la Princesa”.

Human postoperative brain tissue was obtained from 23 patients suffering intractable TLE. The resection of the neocortex and the amygdalo-hippocampal area, tailored according to the electrocorticography findings, was performed as described previously [19]. Immediately after removal, biopsy samples were fixed in cold 4% paraformaldehyde and small blocks (∼15×10×10 mm) were obtained that covered the entire rostrocaudal extent of the hippocampal formation. These blocks were immersed in a solution of 4% paraformaldehyde in 0.1 M phosphate buffer (pH 7.4) for 24–36 h. at 4°C. Serial coronal vibratome sections (50 µm) were then obtained from these blocks. Histological analysis was performed in all cases (for details, see Text S1).

From the cohort of 260 epileptic patients from this hospital, we selected patients with unilateral TLE and showing well-defined hippocampal sclerosis (n = 39; confirmed by histopathological analysis after surgery). From this subgroup, only subjects fulfilling the following inclusion criteria were included (n = 23): older than 16 years old on the date of surgery; well-defined lateralization by video electroencephalography; unilateral hippocampal sclerosis suggested by MRI; over 2 years of postoperative follow up; and a complete neuropsychological evaluation (that included an assessment of cognitive-perceptive and emotional processes).

In the present study, the clinical feature “Side” refers to the side associated with seizure onset as determined by video-EEG studies. These patients all suffered from partial complex and secondarily generalized seizures (age range, 17–54 years; average, 34.3 years; age of onset range, 0.7–20 years; average, 9.63 years; duration range, 4–40 years; average, 24.96 years; Table 1).

**Table 1:** Summary of the clinical data from the epileptic patients and the surgical outcome.

Patient	Age (years),sex, side	Age of onset,duration (years)	Seizure type	Seizure frequency	Engel scale for surgicaloutcome/years after surgery
H48	41, m, L	18, 23	gen	weekly	I/12
H57	27, m, R	13, 14	PC	3 weekly	I/11
H61	17, f, R	7, 17	PC	2 weekly	I/11
H67	39, m, R	1, 38	gen	weekly	I/11
H75	37, m, L	13, 24	PC	2 weekly	II/10
H84	31, m, R	2, 29	gen	4 weekly	I/10
H94	27, m, L	20, 7	gen	3–5 weekly	II/9
H104	32, m, L	12, 20	PC	weekly	I/9
H108	50, m, L	15, 35	gen	4 weekly	III/9
H109	22, f, R	4, 18	PC	0–3 weekly	I/9
H115	40, f, L	1.8, 38	gen	4 weekly	III/9
H123	24, f, L	7, 17	gen	daily	I/8
H136	20, f, R	0.7, 19	gen	weekly	I/8
H220	53, f, L	13, 40	PC	weekly	I/4
H225	49, f, R	16, 33	PC	weekly	I/4
H229	40, f, R	2, 38	PC	weekly	I/4
H230	22, f, L	18, 4	PC, gen	daily	III/3
H231	23, m, L	1, 22	PC, gen	daily	I/3
H233	35, m, L	6, 29	PC, gen	weekly	III/3
H236	54, f, R	16, 38	PC, gen	weekly	I/3
H237	41, f, L	20, 21	PC, gen	2 weekly	I/3
H238	22, f, R	11, 11	PC	weekly	I/3
H241	43, f, L	4, 39	PC, gen	Not regular	I/3

f: female, gen: secondarily generalized, L: Left, m: male, PC: partial complex seizures, PS: partial simple seizures, R: right, Engel scale for surgical outcome: class I seizure-free, class II rare seizures and class III worthwhile improvement.

### Neuropsychological Testing

Pre-surgical evaluation of patients was conducted in order to examine the relationship between cognitive impairment and neurological damage. Intelligence was assessed using the Wechsler Adult Intelligence Scale-Third Edition (WAIS-III) [20], which provides a verbal intelligence quotient (VIQ), a performance intelligence quotient (PIQ), and a full scale intelligence quotient (FSIQ). In the present study, intelligence quotients (IQ) were categorized into seven groups, depending on the values obtained for each index (in parenthesis): Very Low (0–69), Low (70–79), Normal-Low (80–89), Normal (90–109), Normal-High (110–119), High (120–129) and Very High (≥130). Patients were classified according to their index values (Table 2).

**Table 2:** Set of predictive variables and their associated values. Cardinalities of each value are shown between brackets. Missing values are also indicated.

**Clinical features:**	**Gender**: Male (10), Female (9)
	**Side**: Left (10), Right (9)
	**Surgery age**: 17- 32 (10), 33- 54 (9)
	**Onset age**: 0- 1 (4), 2- 10 (7), 11- 20 (8)
	**Elapsed time**: 7- 13 (3), 14- 19 (4), 20- 39 (12)
	**Type of seizure (SeizureType)**: Generalized (8), Partial Complex (7) or Both (4)
	**Frequency of seizures (SeizureFreq)**: Daily (2), Weekly (8), 2-Weekly (2), 3-Weekly (3), 4-Weekly (3), Other (1)
	**Febrile seizures (Febrile)**: Negative (10), Positive (9)
**Neuropsychological features:**	**VIQ**: Low (1), Normal-Low (4), Normal (8), Normal-High (3), High (1) - missing (2)
	**PIQ**: Low (1), Normal-Low (1), Normal (11), Normal-High (3), High (1) - missing (2)
	**FSIQ**: Low (0), Normal-Low (4), Normal (9), Normal-High (3), High (1) - missing (2)
	**MlogI**: Low (2), Normal-Low (5), Normal (11), Normal-High (0), High (0) - missing (1)
	**MlogII**: Low (2), Normal-Low (3), Normal (13), Normal-High (0), High (0) - missing (1)
	**MvisI**: Low (0), Normal-Low (2), Normal (13), Normal-High (1), High (2) - missing (1)
	**MvisII**: Low (0), Normal-Low (6), Normal (9), Normal-High (1), High (2) - missing (1)
	**Schizophrenia index**: Negative (9), Positive (3) - missing (7)
	**Coping deficit index**: Negative (5), Positive (7) - missing (7)
	**Depression index**: Negative (4), Positive (8) - missing (7)
	**P. Style**: EB1 (3), EB2 (6), EB3 (3) - missing (7)

To examine possible dysfunction in the hippocampus and neocortex, individuals were assessed using the Wechsler Memory Scale (WMS) [21]. Specifically, we performed logical and visual memory measurements for both immediate (immediate logical memory, MlogI; immediate visual memory, MvisI) and delayed recalls (delayed logical memory, MlogII; delayed visual memory, MvisII). In all cases, the patients were classified into five groups (Table 2) based on the WMS index (indicated in parenthesis): Low (50–65), Normal-Low (66–80), Normal (81–115), Normal-High (116–130) and High (≥131).

The Rorschach test was used to evaluate the cognitive-perceptive and emotional processes that occur during the individual response, with no interference of language understanding or cultural variables [14]. The Rorschach test was applied following the comprehensive system [14], [22]–[23]. Rorschach evaluation requires specific training and clinical experience and is not always included in the neuropsychological battery. In the present study, Rorschach test was applied by Dr. Jesús de Felipe-Oroquieta, who is a specialist in clinical psychology, neuropsychology and in Rorschach Test.

Only certain variables of the Rorschach test were analyzed, according to the psychopathology of the epileptic patients, namely: the schizophrenia index (Sczi), social ability index (Cdi) and depression index (Depi). To evaluate problem resolution and decision making in which emotionality and ideation are involved, the personality style of all patients was assessed. The personality style (P. Style), or erlebnistypus (EB), is a relevant variable in the Rorschach test, which quantifies the cognitive-perceptive responses of the subject [14]. The responses to perception of movement of introversive subjects are two points higher than their responses to weighted color, while extratensive subjects are those whose responses to color are greater than their responses to movement. Finally, ambitents are individuals for whom the difference between the two variables is less than two points. Thus, patients were classified as introversive (EB1), extratensive (EB2) or ambitent (EB3). The Rorschach protocols followed were those included in the Rorschach® Interpretation Assistance Program (RIAP5: John E. Exner Jr., Irving B. Weiner, and Par Staff; Psychological Assessment Resources Inc. Lutz, FL, USA). In order to group patients, the Sczi, Cdi and Depi indices were evaluated and classified as positive or negative (Table 2).

### Supervised Classification Data Mining

The data was analyzed using supervised classification techniques. This design treats the feature defining the problem differently (as either full recovery from epilepsy or not). This variable is usually termed the class variable. Patient outcome was evaluated after surgery using the Engel's scale [24]: Class I, seizure free (n = 14); Class II, rare disabling seizures (almost seizure free; n = 2); Class III, worthwhile improvement (n = 3). Classes II and III reflect improvement in the disease but not complete recovery. For this reason, both categories were grouped together. Thus, the supervised class variable describes seizure free patients (n = 14) and those exhibiting an improvement only (n = 5). Finally, the missing values mentioned in Table 2 were entered using the mode of the variable with missing values conditioned to the class variable.

Three different classification paradigms were used in the experiment: naïve Bayes, logistic regression with ridge estimators and k-nearest neighbor (k-NN). To assess the classification performance of each paradigm, we used a leave-one-out cross-validation (LOOCV) scheme. This validation scheme estimates the accuracy of a given classification model by inducing the same number of classifiers as comprises the dataset. Each intermediate model is built using all instances but one, and tested on the excluded instance. The accuracy estimation of the final model is obtained as the average accuracy of all intermediate classifiers. In addition to the estimated classification accuracy, the area under the receiving operating characteristic (ROC) curve or area under the curve (AUC) [25] was also calculated. These two measurements are classically used to compare classification models (for further details see Text S2).

### Feature Subset Selection

To determine the best subset of features for classification and analyze the relevance of each feature, we developed a robust feature subset selection procedure to remove irrelevant and/or redundant features that hindered the classification procedure. Given the limited number of samples in the dataset, we approached the selection process by producing similar datasets of equivalent sizes but with slight differences. A total of 1,000 intermediate datasets were produced by random stratified resampling with replacement of the original dataset. For each dataset, a subset of features was selected using a race search algorithm [26].

Race search, or racing, applies paired and unpaired t-tests to the cross-validation accuracies of competing subsets of features. When a significant difference exists between the means of the errors of two competing subsets, the poorer of the two can be eliminated from the race. The racing search was configured as a backward elimination, whereby the initial subset included all the original features and individual deletions were performed until no improvement was detected. For consistency in all experiments, a LOOCV scheme was used again. Once all the selections had been performed, the frequencies of each feature were computed as the number of times it was included in the model. In total, three frequency rankings were used, one for each classification paradigm. To determine which features to retain for each classification paradigm in the final model, a LOOCV scheme was applied adding features from those most often selected to those selected least. Similar procedures have achieved remarkable results when applied to similarly problematic datasets [27]–[29].

## Results

### Dataset Compilation

Out of the 23 subjects selected, no behavioral testing data was available for 4 subjects. Since this implies that many variables are lost in the dataset, these four samples were not used in the study. Accordingly, the data mining task was performed with 19 samples. The database comprised 19 features or variables, belonging to two different groups: 8 clinical variables and 11 items derived from neuropsychological tests (the number of samples per feature and value is shown in Table 2).

### Analysis of Pre-surgical Features

The data was analyzed using supervised classification techniques. The class feature refers to the Engel's scale value indicated [24], grouped into two categories: seizure free (Engel grade I) or improvement only (Engel grades II and III). The classification was constructed using 19 variables, based on measurements taken before surgery (Table 2).

**Figure 1:**
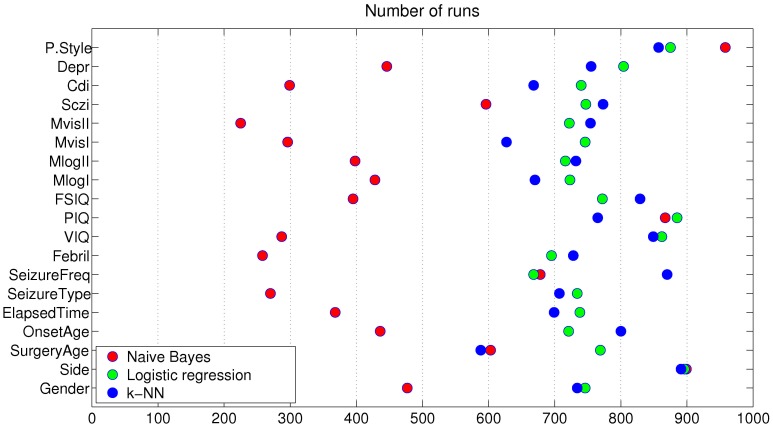
Number of times that features were included in the different intermediate subsets selected by the race search feature selection for over 1,000 dataset resamplings.

**Figure 2:**
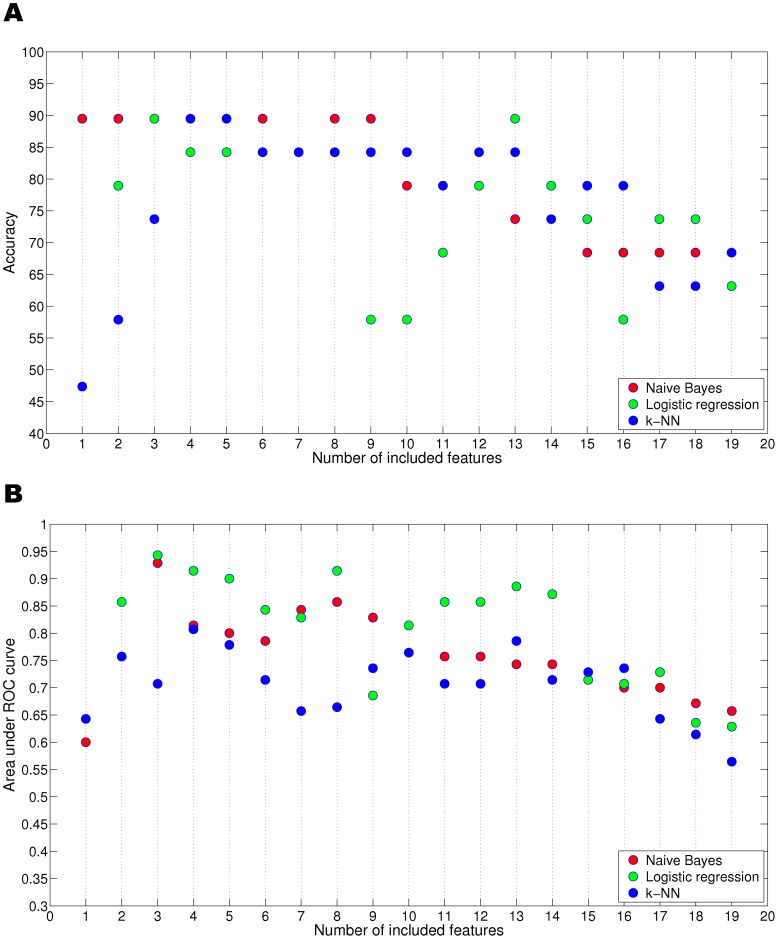
Estimated classification performance using LOOCV validation. Only features available before surgery were included in this performance analysis. The x-axis reflects the size of the subset of features retained. A) The upper chart shows the estimated accuracy; whereas, B) the lower chart shows the associated area under the ROC curve. Note that the features for a given point on the x-axis can differ depending on the classifier used (see Table 3 for the respective feature subsets).

First, we determined the frequencies of the features selected during the search process in order to identify the variables (or subsets of variables) most relevant to the analysis (Figure 1). Choosing a robust and reliable set of relevant features is crucial, as the dimensionality of the database may overfit the results. To avoid such an effect, we performed a series of tests to analyze the classification power of the different subsets of features. Using a top-down scheme, we added features iteratively, one at a time, according to the frequency ordering obtained by the relevance analysis. Thus, the addition of features was guided by the ranking associated with each feature (as shown in Table 3). Accordingly, the first database projection was composed of only one feature, which was selected most frequently during the race selection. This process was repeated until all the features were included. The subsets of selected features in each race were not always the same and hence, although they are presented together for simplicity in Figure 2, the subsets that correspond to the same number of features (X axis) were almost always different. The naïve Bayes model tends to select subsets with fewer features than those selected by either logistic regression or k-NN. Both logistic regression and k-NN are more stable in their behavior, with selection frequencies ranging from 600 and 900 for all variables. Some divergence was observed within the selection. For instance, PIQ was selected less than 300 times by the naïve Bayes model, but approximately 850 times by the other two classifiers. Nevertheless, three features were consistently selected by the three classifiers with high frequency, namely Side, PIQ and P. Style (Table 3).

**Table 3:** Variables ordered according to their frequency of selection during resampling and variable subset selection (occ: occurrence). Three different rankings are displayed - one for each of the classifiers used during the search.

Ranking	Naïve Bayes		Logistic regression		k-NN	
	Variable	occ.	Variable	occ.	Variable	occ.
1	P. Style	958	Side	896	Side	891
2	Side	899	PIQ	885	SeizureFreq	870
3	PIQ	867	P. Style	875	P. Style	857
4	SeizureFreq	678	VIQ	862	VIQ	849
5	SurgeryAge	603	Depi	804	FSIQ	829
6	Sczi	596	FSIQ	772	OnsetAge	800
7	Gender	477	SurgeryAge	769	Sczi	773
8	Depi	446	Sczi	747	PIQ	765
9	OnsetAge	436	Gender	746	Depi	755
10	MlogI	428	MvisI	746	MvisII	754
11	MlogII	398	Cdi	740	Gender	734
12	FSIQ	395	ElapsedTime	738	MlogII	732
13	ElapsedTime	368	SeizureType	734	Febrile	728
14	Cdi	299	MlogI	723	SeizureType	707
15	MvisI	296	MvisII	722	ElapsedTime	699
16	VIQ	287	OnsetAge	721	MlogI	670
17	SeizureType	270	MlogII	716	Cdi	668
18	Febrile	258	Febrile	695	MvisI	627
19	MvisII	225	SeizureFreq	668	SurgeryAge	588

For each of the projected datasets, the performance of the associated classifier was estimated using an LOOCV scheme to determine the classification power of the different feature subsets.

When naïve Bayes and logistic regression were used with three features, 89.47% accuracy was achieved in both cases with AUC values of 0.9285 and 0.9428, respectively, and an identical F-measure value of 0.9333 was obtained (Figure 2). The subset of three features was the same in both cases and comprised Side, P. Style and PIQ (Table 3). The best accuracy using the k-NN approach (again 89.47%, AUC value = 0.8071 and F-measure = 0.9035) was achieved using four features, namely Side, P. Style, VIQ and Frequency of seizures.

As the three models used achieved the same level of estimated accuracy (89.47%), there was no need to choose between them. Moreover, the improvement with respect to the analyses including all the variables was significant; the inclusion of all 19 variables (Figure 2 and Table S3) resulted in accuracies of 68.42%, 63.15% and 68.42%, for the naïve Bayes, logistic regression and k-NN, respectively (with AUC values ranging between 0.5642 and 0.6571, and F-measures between 0.7404 and 0.8125: Figure 2).

### Hypothesis Test on the Pre-surgical Data

As a complementary measure, we performed a classical Wilcoxon signed-rank hypothesis test on the values obtained for all features in the classification. This test is a non-parametric statistical hypothesis test, in which the null hypothesis (H0) states that two samples come from a distribution with the same median. In our case, the two samples are the sets of each feature values grouped using the Engel output. Considering again the class variable (Engel) as the grouping variable, the features for which the null hypothesis (H0) is rejected will constitute the relevant features for the classification. Three features presented significant values with a 95% confidence level: Side, PIQ and P. Style (Table 4), confirming their importance. Values of these features –stratified by class– are as follows: Seizure free (50% Left and 100% Right Side; 73.7% Normal, 100% Normal-High, 100% High PIQ; 100% EB1, 84.6% EB2 P. Style), and, Only improvement (50% Left Side; 100% Low, 100% Normal-Low, 26.3% Normal PIQ; 15.4% EB2, 100% EB3 P. Style). The combination of their values using both the naïve Bayes and the logistic regression reached maximum accuracy, further supporting our findings.

**Table 4:** Features and associated p-values obtained from a Wilcoxon signed-rank test comparing the values of each feature with the Engel output. in order of increasing p-value.

Feature	p-value
P. Style	0.0134†
Side	0.0433†
PIQ	0.0492†
SeizureFreq	0.1471
FSIQ	0.2334
Surgery Age	0.2384
Sczi	0.3096
Gender	0.3684
MvisI	0.4226
Depi	0.5165
MvisII	0.6022
MlogII	0.6070
Onset Age	0.6140
SeizureType	0.6140
ElapsedTime	0.7322
VIQ	0.8168
MlogI	0.8894
Cdi	1
Febrile	1

† indicates statistical significance at a 95% confidence level. Features are listed.

### Post-surgery Analysis

After surgery, the eleven psychological features were re-evaluated in the cohort of patients, although some values were lost due to the practical difficulties in assessing all patients. In addition, schizophrenia was removed as a feature, as it was irrelevant since there were no such cases. Therefore, 30 values (out of 165) were assigned using the associated conditional modes (based on the value of the Engel output) and, in total, there were 10 attributes and 15 instances: seizure-free (10) and improvement only (5).

The effects of surgery on cognitive function were first analyzed by comparing the psychological test results before and after surgery. The simplest way to compare these values was to subtract the post-surgery values from the pre-surgery values (the resulting values are denoted with the prefix Δ in Table S1). When these differences were compared using the Wilcoxon test, no significant differences were evident for any of the psychological features studied.

To investigate the possible link between post-surgery psychological evaluation scores and surgical outcome, we performed a cluster analysis of all cases with respect to the post-surgery variables. A multinomial mixture model in which the parameters were estimated using the expectation-maximisation (EM) algorithm was selected for this purpose. This algorithm provides the probability that a given case belongs to a cluster rather than a hard assignment to that cluster. Since the distribution of the cases in terms of the Engel output was already known, the groups reported by the algorithm could be validated. This validation was satisfactory and when two groups were created, 13 out of the 15 cases were correctly clustered. Of the 13 correctly grouped cases, 8 were clustered in one group, while the remaining 5 were clustered in a second group along with the 2 incorrectly clustered cases (Table 5). This separation implies a significant pattern in post-surgical psychological evaluations that may be linked to surgery. This is more remarkable in the cases of "seizure-free" output, which maps with the first cluster.

**Table 5:** Probabilities of belonging to cluster 0 or cluster 1 for each case.

Case	p(c_0_|x)	p(c_1_|x)	Engel
1	0.08417	0.91583	s1-seizure-free†
2	0.98861	0.01139	s1-seizure-free
3	0.95076	0.04924	s1-seizure-free
4	0.06282	0.93718	s2_3-only improvement
5	0.59592	0.40408	s1-seizure-free
6	0.01103	0.98897	s2_3-only improvement
7	0.99529	0.00471	s1-seizure-free
8	0.0332	0.9668	s2_3-only improvement
9	0.5379	0.4621	s1-seizure-free
10	0.01391	0.98609	s2_3-only improvement
11	0.00381	0.99619	s1-seizure-free†
12	0.95596	0.04404	s1-seizure-free
13	0.93247	0.06753	s1-seizure-free
14	0.5171	0.4829	s1-seizure-free
15	0.00944	0.99056	s2_3-only improvement

The last column shows the actual Engel score (not used in the clustering).

† indicates cases that were incorrectly clustered on the basis of the probability assigned by the clustering algorithm.

We compared the post-surgery values of each feature with regard to the clustering assignments produced by the algorithm. It should be noted that the correspondence between the EM assignment and the real Engel outcome was not perfect (2 errors, Table S2). This analysis revealed significant differences for three features: postPIQ, postFIQ and postMvisII. Thus, the values of these three variables would appear to be relevant to the grouping differences between the cases in each cluster. Unfortunately, these results are of limited use because the association variable in the test is not actually adjusted to the class variable itself. We can only state that there are significant differences in these three features between the two clusters.

## Discussion

Epileptic patients with pharmacoresistant TLE that are candidates for surgery are evaluated using time-consuming and expensive tests. Although epilepsy surgery is effective in reducing both the number and frequency of seizures, a significant proportion of these patients continue to suffer seizures after surgical intervention. Accordingly, there is considerable interest in identifying predictors of the surgical outcome in patients with TLE (for recent reviews see [10]–[11]). However, to the best of our knowledge, the relevance of individual clinical and psychological features has not yet been studied in detail, nor has a Rorschach evaluation of individual personality been included in the standard neuropsychological assessments used to predict surgical outcome [30]. The complexity of the data obtained from epileptic patients means it must be analyzed in a specific manner to identify any relationships and patterns. Given the usefulness of data mining in medical applications [31], we used machine learning tools to evaluate the ability of three supervised classifiers (naïve Bayes, logistic regression and k-nearest neighbor) to predict the outcome of epilepsy surgery.

### Analysis of the Pre-surgical Features

The first stage of our analysis involved the use of supervised classification to select features. Since all the features were included in the classification, this led to the generation of irrelevant and/or redundant information and we found that using three specific features produced the most accurate results: Side, P. Style and PIQ. Since locating the side on which seizure activity originates is generally accurate, particularly when determined by video electroencephalography [8]–[9], this feature represents a clear predictive variable. However, the findings also emphasize the importance of psychological features as good predictors (P. Style and PIQ).

Analysis of personality style revealed that while ambitent style (EB3) was associated with a poor surgical outcome (Engel II–III), introversive patients (EB1) had better surgical outcomes (Engel I) and that the outcome in the majority (85%) of extroversive (EB2) patients was good (Engel I). It is plausible that as yet unknown and complex mechanisms in the brain may underlie the relationship between different personality styles and the benefits that surgery may produce. PET studies performed on TLE patients previously evaluated with a Rorschach test have demonstrated predominant hypometabolism in the left hemisphere of introversive patients, while extratensives displayed hypometabolism in the right hemisphere [32]. However, the cortical circuits involved in personality style and their role in seizure activity associated with hippocampal sclerosis remain unclear.

Patients with a normal-to-high and high PIQ displayed good surgical outcomes (Engel I), whereas seizure frequency was not reduced significantly in patients with low or normal-to-low PIQ scores (Engel II–III). Moreover, in the normal PIQ subgroup, a large proportion of the patients (77%) became seizure free (Engel I). The cognitive processes involved in the performance intelligence test remain unknown, although brain imaging studies suggest that some cortical regions are more critically involved than others, such as the frontal lobes [33]–[34]. In addition, lower PIQ may be associated with severe epilepsy, and perhaps with widespread brain disturbances, thus, the response to treatment would be more limited. Moreover, a positive correlation has been reported between PIQ scores and cortical grey matter thickness in regions of the temporal cortex [35]–[36]. Reduced cortical thickness, as measured by MRI, has also been described in the temporal, parietal, occipital and frontal lobes of TLE patients with hippocampal sclerosis [37]. Unfortunately, no volumetric cortical MRI studies were performed in our series of patients, precluding any analysis of the correlation between cortical volume and PIQ scores.

The three classification paradigms used in the present study were equally effective as tools to select epileptic patients as surgery candidates, with each showing the same estimated accuracy (89.47%). This estimation could be slightly overoptimistic as a side effect due to the use of LOOCV. However, our results revealed that relevant features are systematically found by all the classification models, reporting the same estimated performance. Even when there is a regularization penalty in the case of logistic regression, particular results are fully consistent with the rest. Therefore, in cases where data is available for the three key variables (Side, PIQ and P. Style) for a given population of TLE patients, any of these three classifiers would be useful when selecting the patients for surgery. Evaluations of similar classifiers with the inclusion of all features penalize the outcome prediction, most likely due to irrelevant and/or redundant information. This fact is in agreement with the behavior of classification models, which is not necessarily monotonic with respect to the inclusion of additional features [38].

Lastly, we found no significant difference in the evaluation of psychological features of our epileptic patients before and after surgery, in line with the idea that personal psychological features are unchanged regardless of surgical outcome.

Although relatively few cases were examined, (n = 19), our findings were verified across all data, suggesting that the machine learning analysis described may become a powerful tool to be included in standard evaluations for epilepsy surgery centers. Since there are several thousand candidates for epilepsy surgery worldwide (with an estimated 100,000 to 200,000 potential candidates in the United States in 2003 alone [7]), the usefulness of our approach could be validated if implemented as a standard test for presurgical evaluation. This external validation on larger cohorts of patients is thus envisaged as crucial future work.

## Supporting Information

Table S1 Wilcoxon signed-rank test comparing post-and pre-surgery values of each feature (denoted with a Δ prefix).
(DOC)

Table S2 Wilcoxon signed-rank test comparing the values of each psychological feature post-surgery using the clustering assignment as the grouping variable.
(DOC)

Table S3 Confusion matrices of the final classification using all features and the most relevant three (naïve Bayes and logistic) and four (k-NN) ones, respectively.
(DOC)

Text S1 Histopathological analyses protocol.
(DOC)

Text S2 Supervised classification paradigms and performance measurement.
(DOC)
