# Supplementary material for: Machine Learning Approach for the Outcome Prediction of Temporal Lobe Epilepsy Surgery
Source: PLoS One. 2013 Apr 30;8(4):e62819. doi: 10.1371/journal.pone.0062819 (PMC3640010; doi:10.1371/journal.pone.0062819)
Supplement: Table S1 — Wilcoxon signed-rank test comparing post-and pre-surgery values of each feature (denoted with a Δ prefix). (DOC) [file pone.0062819.s001.doc]

***Table S1***. Wilcoxon signed-rank test comparing post-and pre-surgery values of each feature (denoted with a Δ preﬁx). Engel output was used as the grouping variable for the test. Values are listed in order of increasing p-value.

| Feature | p-value |
| --- | --- |
| ΔMvisI | 0.3297 |
| ΔVIQ | 0.3357 |
| ΔMvisII | 0.4868 |
| ΔDepi | 0.5714 |
| ΔCdi | 1 |
| ΔFSIQ | 1 |
| ΔMlogI | 1 |
| ΔMlogII | 1 |
| ΔPIQ | 1 |
| ΔP. Style | 1 |
