# Supplementary material for: Machine Learning Approach for the Outcome Prediction of Temporal Lobe Epilepsy Surgery
Source: PLoS One. 2013 Apr 30;8(4):e62819. doi: 10.1371/journal.pone.0062819 (PMC3640010; doi:10.1371/journal.pone.0062819)
Supplement: Table S2 — Wilcoxon signed-rank test comparing the values of each psychological feature post-surgery using the clustering assignment as the grouping variable. (DOC) [file pone.0062819.s002.doc]

***Table S2***. Wilcoxon signed-rank test comparing the values of each psychological feature post-surgery using the clustering assignment as the grouping variable. † indicates statistical signiﬁcance at α =0.05. Values are listed in order of increasing p-value.

| Feature | p-value |
| --- | --- |
| MvisII | 0.0112† |
| PIQ | 0.0392† |
| FSIQ | 0.0460† |
| P. Style | 0.1399 |
| MvisI | 0.2480 |
| MlogI | 0.4115 |
| Depi | 0.4615 |
| MlogII | 0.8531 |
| Cdi | 0.8923 |
| VIQ | 0.9054 |
