# Supplementary material for: Machine Learning Approach for the Outcome Prediction of Temporal Lobe Epilepsy Surgery
Source: PLoS One. 2013 Apr 30;8(4):e62819. doi: 10.1371/journal.pone.0062819 (PMC3640010; doi:10.1371/journal.pone.0062819)
Supplement: Table S3 — Confusion matrices of the final classification using all features and the most relevant three (naïve Bayes and logistic) and four (k-NN) ones, respectively. (DOC) [file pone.0062819.s003.doc]

***Table S3***. Confusion matrices of the final classification using all features and the most relevant three (naïve Bayes and logistic) and four (k-NN) ones, respectively. Rows correspond to actual values whereas columns display the predictions by the classifiers. Engel output is encoded as GI for seizure-free and GII-III for improvement only.

| Naïve Bayes | | |  | Logistic regression | | |  | k-NN | | |
| --- | --- | --- | --- | --- | --- | --- | --- | --- | --- | --- |
|  | | |  |  | | |  |  | | |
| *All features* | | |  | *All features* | | |  | *All features* | | |
|  | GI | GII-III |  |  | GI | GII-III |  |  | GI | GII-III |
| GI | 11 | 3 |  | GI | 10 | 4 |  | GI | 13 | 1 |
| GII-III | 3 | 2 |  | GII-III | 3 | 2 |  | GII-III | 5 | 0 |
|  |  |  |  |  |  |  |  |  |  |  |
| *{Side, PIQ, P.Style}* | | |  | *{Side, PIQ, P.Style}* | | |  | *{Side, P.Style, SeizureFreq}* | | |
|  | GI | GII-III |  |  | GI | GII-III |  |  | GI | GII-III |
| GI | 14 | 0 |  | GI | 14 | 0 |  | GI | 14 | 0 |
| GII-III | 2 | 3 |  | GII-III | 2 | 3 |  | GII-III | 5 | 0 |
|  |  |  |  |  |  |  |  |  |  |  |
| *{Side, PIQ, P.Stlye, SeizureFreq}* | | |  | *{Side, PIQ, P.Style, VIQ}* | | |  | *{Side, P.Style, VIQ, SeizureFreq}* | | |
|  | GI | GII-III |  |  | GI | GII-III |  |  | GI | GII-III |
| GI | 14 | 0 |  | GI | 13 | 1 |  | GI | 14 | 0 |
| GII-III | 2 | 3 |  | GII-III | 2 | 3 |  | GII-III | 2 | 3 |
