# Supplementary material for: Machine Learning Approach for the Outcome Prediction of Temporal Lobe Epilepsy Surgery
Source: PLoS One. 2013 Apr 30;8(4):e62819. doi: 10.1371/journal.pone.0062819 (PMC3640010; doi:10.1371/journal.pone.0062819)
Supplement: Text S1 — Histopathological analyses protocol. (DOC) [file pone.0062819.s004.doc]

**Histopathological analyses**

Standard neuropathological assessment of the surgically removed tissue demonstrated the existence of hippocampal sclerosis in all patients (n=23) [1-2]. Sections of the hippocampal formation were batch-processed using Nissl-staining and standard immunocytochemical techniques. Free-floating sections were pretreated in 1% H_2_O_2_ for 30 min to remove endogenous peroxidase activity, and then for 1 h. in PB with 0.25% Triton-X (Merck, Darmstadt, Germany) and 3% normal horse serum (Vector Laboratories, Burlingame, CA, USA). The sections were then incubated overnight at 4°C with an antibody against a mouse neuron specific nuclear protein (NeuN, 1: 2000: Chemicon, Temecula, CA, USA). Antibody binding was detected with the avidin-biotin method, using a secondary horse anti-mouse biotinylated antibody (1:200: Vector Laboratories) and using the Vectastain ABC immunoperoxidase kit (Vector Laboratories) and 3,3’-diaminobenzidine tetrahydrochloride (DAB, Sigma-Aldrich, St. Louis, MO, USA) as the chromogen. The sections were mounted, dehydrated, cleared with xylene and coverslipped.

**Supporting Information References**

1. Arellano JI, Ballesteros-Yanez I, DeFelipe J, Munoz A, Sola RG (2004) Histopathology and reorganization of chandelier cells in the human epileptic sclerotic hippocampus. *Brain* 127:45-64.

2. Kastanauskaite A, Alonso-Nanclares L, Blazquez-Llorca L, Pastor J, Sola RG, et al. (2009) Alterations of the microvascular network in sclerotic hippocampi from patients with epilepsy*.* *J Neuropathol Exp Neurol* 68:939-950.
