# Supplementary material for: Machine Learning Approach for the Outcome Prediction of Temporal Lobe Epilepsy Surgery
Source: PLoS One. 2013 Apr 30;8(4):e62819. doi: 10.1371/journal.pone.0062819 (PMC3640010; doi:10.1371/journal.pone.0062819)
Supplement: Text S2 — Supervised classification paradigms and performance measurement. (DOC) [file pone.0062819.s005.doc]

**Supervised classification paradigms**

Data was analyzed using supervised classification techniques, a design that treats the separation feature of the problem distinctly (full recovery from epilepsy or not), usually termed the class variable. Thus, the class variable is the previously mentioned Engel's scale grouped into two states: seizure free or improvement only. Three different classification paradigms were used, as described below:

• Naïve Bayes [1] is based on the assumption of conditional independence between the predictor variables given the class. The model parameters are estimated based on the maximum likelihood estimators.

• Logistic regression [2] is based on the logistic function and allows an interpretation in terms of probability. A set of parameters is estimated from the training data, usually known as regression coefficients. Usually, regression coefficients are estimated using the maximum likelihood estimation method, although adaptations exist that penalize this maximum likelihood with other factors. The logistic regression model used in this study penalizes the likelihood function by shrinking the regression coefficients with an L2 norm (ridge regression, [3]).

• The k-nearest neighbor (k-NN) algorithm [4] classifies in terms of similarity: unlabeled examples are classified based on their distance from the examples in the training set. As k-NN has no explicit classification model, there is no learning stage. The k-NN finds the k closest cases in the data and assigns the unlabeled case to the class that most frequently appears within the k-subset. For our experiments, k-NN was computed with Euclidean distance and a k value of three to avoid ties.

To assess the performance of each of the classification paradigms, we used a leave-one-out cross-validation scheme (LOOCV). This validation scheme estimates the accuracy of a given classification by inducing the same number of classifiers as comprises the dataset. Each classifier is induced using all the instances except one, ensuring that the instance that is left out is different each time. The classifiers are then tested against this 'left-out' instance and the result is stored. Finally, the accuracy estimation of the entire process is computed as the average of all the tests. The LOOCV scheme is known to produce unbiased accuracy estimations [5], although it can return optimistic errors in some domains. Nevertheless, due to the small size and unbalanced class distribution of our dataset, this validation scheme was expected to provide realistic values. The LOOCV estimation could be potentially damaged by changes in the training set; i.e. the variance term in the estimation is the problematic part. In contrast, LOOCV estimators are considered to be unbiased. Braga-Neto has discussed this in various papers showing that there is no “perfect” estimator when the number of cases is low [6]. He found no statistical differences between repeated cross validations and LOOCV.

One of the common reasons to choose one over another is the computational burden. LOOCV is often discarded since it is computationally demanding as the number of cases increase. In our case, bearing in mind our low number of cases, we decided to use it as our measure of classification performance. This choice was used for all the validations done in the data mining analysis, in order to maintain internal consistency.

Over-optimistic estimations might be important when comparing different classification paradigms [7]. LOOCV is used as a tool to guide the race track search and to compare the estimations when the number of features varies using the same classification model. We found coherent results in the estimated accuracy, the number of features and which these features are across all classifiers.

In addition to the estimated classification accuracy, the area under the ROC curve or AUC [8] was also considered. In dichotomic problems, classification performance can be visually inspected by presenting the confusion matrix. In the classical set-up, each column gathers the number of instances classified as either Positive or Negative. The rows indicate how many of these classifications are based on reality or the actual class label, and how many are not.

The main diagonal values in a confusion matrix correspond to the correctly classified instances, which are the number of true positives (TP) and the number of true negatives (TN). The misclassification values are divided into false negatives (FN) and false positives (FP), depending on the direction of the mistake. The classifier’s accuracy corresponds to the ratio between the sum of TP and TN, and the total number of instances classified. However, other specific measures are available, such as sensitivity and specificity values.

The sensitivity (Sen) is the ratio of positive instances that are correctly classified as positive, also known as recall (r) or true positive rate (TPR), and it is computed as:

Sen = r = TPR = TP / (TP + FN).

The counterpart of sensitivity for the negative instances is the specificity (Spe), also known as false negative rate (FNR). This is the ratio of actual negative cases that are correctly classified, and is calculated as:

Spe = FNR = TN / (TN + FP).

Finally, the receiver operating characteristic (ROC), or simply ROC curve, is a plot of the sensitivity (TPR) versus the false positive rate (1-Spe or 1-FNR) for a binary classifier system as its discrimination threshold varies. The AUC is the area that this curve covers and is a classical measure used to compare classification models.

The estimated accuracy for all the classifiers using all the features was quite poor, ranging from 63.16% - 68.42% (0.564-0.657 in AUC). In the search for a better classification performance, we devised a feature subset selection procedure to reduce the number of features in the hope of removing irrelevant and/or redundant features that lower the accuracy of the classification results. Since the size of the dataset is so small, we approached the selection by producing similar datasets of the same size but with slight differences. In this way, 1,000 intermediate datasets were produced by random resampling with replacement of the original dataset. For each of these datasets, a subset of features was selected using a race search algorithm [9]. Race search, or racing, uses paired and unpaired t-tests on cross-validation accuracies of competing subsets of features. A significant difference between the means of the accuracies of two competing subsets permits the poorer of the two to be eliminated from the race. Since the number of samples is particularly small, the racing search was configured as a backward elimination in which the initial subset included all the original features, and single deletions were performed until no further improvement was observed. To ensure consistency between the experiments, the validation scheme used was the LOOCV. The computing time of this race search algorithm makes it ideal for analysis of all the possible combinations in our data flow analysis. Once all the selections had been made, the frequency for each feature was computed as the number of times it had been included. In total, we obtained three frequency rankings, one for each classification paradigm, with three different frequencies per feature. To select which features to retain for each classification paradigm in the final classification model, an LOOCV validation scheme was used. Features were added according to their frequencies of selection, with those features selected most often taking preference over those that were selected least. The whole pipeline data process was implemented in Java using the Waikato Environment for Knowledge Analysis (WEKA) application programming interface [10].

**Supporting Information References**

1. Minsky M (1961) Steps toward artificial intelligence. *Proceedings of the IRE* 49:8-30.

2. Kleinbaum DG (1994) *Logistic Regression: A Self-Learning Text*. New York: Springer-Verlag.

3. Le Cessie S, Van Houwelingen JC (1992) Ridge estimators in logistic regression. *Applied Statistics* 41:191-201.

4. Aha DW, Kibler D, Albert MK (1991) Instance-based learning algorithms. *Machine Learning* 6:37-66.

5. Lachenbruch P, Mickey M (1968) Estimation of error rates in discriminant analysis. *Technometrics* 10:1-11.

6. Braga-Neto U (2005). Small-sample error estimation: Mythology versus mathematics. *In Proceedings of SPIE, the International Society for Optical Engineering*, 5916:304-314.

7. Rodríguez JD, Pérez A, Lozano JA (2013) A general framework for the statistical analysis of the sources of variance for classification error estimators. Pattern Recognition, 46(3), 855-864.

8. Pepe MS (2004) *The Statistical Evaluation of Medical Test for Classification and Prediction*. Oxford: Oxford University Press.

9. Moore AW, Lee MS (1994) Efficient algorithms for minimizing cross validation error. *Proceedings 11^th^ International Conference on Machine Learning*, 190-198.

10. Hall M, Frank E, Holmes G, Pfahringer B, Reutemann P, et al (2009) The WEKA Data Mining Software: An Update. *SIGKDD Explorations* 11(1):11-18.
